# Predicting Tacit Coordination Success Using Electroencephalogram Trajectories: The Impact of Task Difficulty

**DOI:** 10.3390/s23239493

**Published:** 2023-11-29

**Authors:** Dor Mizrahi, Ilan Laufer, Inon Zuckerman

**Affiliations:** Department of Industrial Engineering and Management, Ariel University, Ariel 4070000, Israel; ilanl@ariel.ac.il (I.L.); inonzu@ariel.ac.il (I.Z.)

**Keywords:** EEG, tacit coordination, trajectories, anonymous random walks, graph embeddings

## Abstract

In this study, we aim to develop a machine learning model to predict the level of coordination between two players in tacit coordination games by analyzing the similarity of their spatial EEG features. We present an analysis, demonstrating the model’s sensitivity, which was assessed through three conventional measures (precision, recall, and f1 score) based on the EEG patterns. These measures are evaluated in relation to the coordination task difficulty, as determined by the coordination index (CI). Tacit coordination games are games in which two individuals are requested to select the same option out of a closed set without the ability to communicate. This study aims to examine the effect of the difficulty of a semantic coordination task on the ability to predict a successful coordination between two players based on the compatibility between their EEG signals. The difficulty of each of the coordination tasks was estimated based on the degree of dispersion of the different answers given by the players reflected by the CI. The classification of the spatial distance between each pair of individual brain patterns, analyzed using the random walk algorithm, was used to predict whether successful coordination occurred or not. The classification performance was obtained for each game individually, i.e., for each different complexity level, via recall and precision indices. The results showed that the classifier performance depended on the CI, that is, on the level of coordination difficulty. These results, along with possibilities for future research, are discussed.

## 1. Introduction

Tacit coordination games refer to social situations in which individuals have to coordinate their actions without explicit communication or shared rules (e.g., [1,2]). These types of problems have been studied mostly in the field of social psychology and economics. The significant value that emerges from analyzing these problems is given by the insights into how individuals can effectively coordinate and reach agreements without the explicit ability to communicate or use shared rules.

Until now, the research combining tacit coordination games with electrophysiological measures was mainly based on spectral analysis (e.g., [3,4,5,6]). Power spectrum analysis is an important part of EEG analysis and enables the extraction of key discriminative features associated with different cognitive processes. In contrast, EEG coherence enables the examination of functional and morphological connections between brain regions [7]. Using these two methodologies, we can provide complementary insights into the dynamics of brain activation and reveal important patterns of neurological activity accompanying tacit coordination.

In one of our recent studies [3], we designed a method that predicts the class label of coherence graph patterns associated with different cognitive states extracted out of multi-channel EEG epochs. Furthermore, in our subsequent study [8], our proposed algorithm found an automated solution such that by analyzing spatial data using random walks [9,10,11], we were able to dramatically improve the classification of the task-related classes associated with precision and recall by ~89% and ~90%, respectively.

The primary goal of this study is to investigate the impact of coordination task difficulty on the ability of our model to accurately predict successful coordination, as reflected in the compatibility of EEG signals between two players engaged in a coordination task. Our study explores tacit coordination using semantic decision making, where the participants align their choices based on the meanings of words, revealing insights into the cognitive processes during collaborative tasks. We hypothesize that the complexity of the coordination task will significantly influence the model’s predictive accuracy, which is a notion supported by findings in the related literature. For instance, it has been observed that stress conditions can lead to decreased cortical network communications in memory-relevant areas, particularly as the task difficulty increases [12]. This indicates that varying task conditions can substantially alter the brain coherence patterns.

To test our hypothesis, we developed a learning model that compares the spatial coherence patterns of two players, represented by graphs derived using the anonymous random walk method [13]. We specifically aim to evaluate the model’s sensitivity—as measured with precision, recall, and the F1 score—against varying levels of task difficulty. The changes in task complexity are expected to affect the brain’s synchronized activities, thereby impacting the model’s ability to achieve accurate predictions. Precision in our model quantifies the accuracy of positive predictions, recall measures the model’s capacity to correctly identify all the instances of coordination, and the F1 score provides a harmonized measure of precision and recall. Together, these metrics offer a comprehensive view of the model’s overall accuracy in differentiating successful coordination under varied task complexities [14].

The evaluation of the model’s performance showed that the difficulty of coordination has a significant effect on the prediction accuracy. Specifically, the results suggested that as the game becomes simpler to coordinate, the precision (the probability of predicting a successful coordination among all the instances predicted as positive) decreases. In contrast, the recall (the probability of predicting successful coordination among all the instances that should have been predicted as positive) increases.

This study connects EEG signals to tacit coordination, unravelling the neural dynamics underlying successful coordination. By analyzing the EEG spatial coherence patterns, we capture coordination’s implicit and synchronized nature beyond explicit communication. Investigating the task difficulty reveals adaptability in challenging situations, enhancing our understanding of coordination. The predictive power of EEG-based classifiers offers practical applications for assessing coordination and introduces avenues for team performance evaluation and human–machine interaction. This integration advances the field, providing fresh insights and paving the way for optimizing coordination strategies. The results of the study are discussed, and recommendations for future work are suggested.

## 2. Materials and Methods

### 2.1. Coordination Index (CI) 

The coordination index (CI) presented in [15,16] is meant to measure the difficulty of a tacit coordination game, that is, the difficulty of choosing the same solution as that of a counterpart player. Measuring the difficulty level is important since it affects both the behavior (e.g., [2]) and the electrophysiological responses (e.g., [17]) of the player. The CI index (see Equation (1)) represents the difficulty of achieving a successful coordination with a score ranging from 0 to 1, where the value 0 represents the maximum coordination difficulty, and 1 represents the easiest coordination level.

(1) $$CI=\frac{\sum_{j}m_{j}(m_{j}-1)}{N(N-1)}$$

where

N—the number of individual players;

$m_{J}$–the number of players who choose solution number #J.

### 2.2. Experimental Design

#### 2.2.1. Single-Epoch Analysis in EEG-Based Coordination Prediction

In our study, each of the ten right-handed participants (six males and four females; mean age: 26.2 years; SD = 3.8) engaged in 12 distinct tacit coordination tasks, during which the EEG data were recorded. The dataset comprises a total of 120 epochs, reflecting a focused analysis approach where each epoch corresponds to a significant moment of decision making within each of the 12 coordination games for every participant. This methodological choice was driven by our study’s objective to evaluate the effectiveness of the model in predicting successful coordination based on the spatial coherence patterns during key decision points. The precision of our model’s predictions hinges on these critical moments when the participants’ decision-making processes are most reflective of their coordination strategy. Each epoch represents a concentrated snapshot of EEG activity, capturing the essence of cognitive processing during these pivotal moments. This approach aligns with the use of the random walk technique in our analysis, which excels in deciphering intricate spatial coherence patterns from these targeted epochs.

Drawing on the established methods in EEG research [18,19], our study adopts a single-epoch approach per participant for each coordination game. This method, which is reflective of the techniques used in other cognitive neuroscience studies, lends credibility to our analytical strategy. By concentrating on individual epochs, we aim to precisely capture the moments of peak cognitive engagement, which is essential for understanding the decision-making processes in coordination tasks. Such an approach ensures that we focus on the most informative instances of brain activity, which might otherwise be diluted in analyses that average over multiple epochs.

#### 2.2.2. Procedure

**Figure 1:**
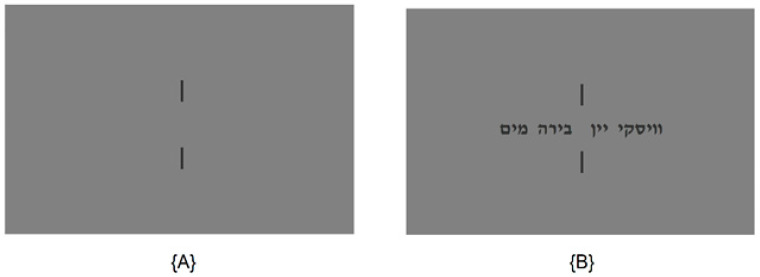
(**A**) Stand by screen. (**B**) Game #1 (“Water”, “Beer”, “Wine”, and “Whisky”).

In each task, the player was presented with four words on a computer screen, some of which had a common denominator (see example in Figure 1). The experimental protocol commenced, with a two-minute resting-state EEG recording, where the participants focused on a red cross on a grey screen to establish a baseline. This was followed by two main experimental stages, each comprising twelve trials with distinct word sets, such as the Hebrew words for “Water”, “Beer”, “Wine”, and “Whisky”. Initially, the participants engaged in a picking task freely selected one word from each set presented. Specifically, the instructions for the picking task were “please pick a single word out of the following set”. In the subsequent coordination task, they aimed to select the same word as an unseen partner from identical sets. Specifically, they were instructed as follows: “Select a single word out of the following list. Your goal is to select the same word that an unknown partner, who is given the same instructions and same list of words, will also select”. To facilitate focus and gaze consistency, each word set was flanked by vertical lines and preceded by a blank slide featuring only these lines, as depicted in Figure 1. 

**Figure 2:**
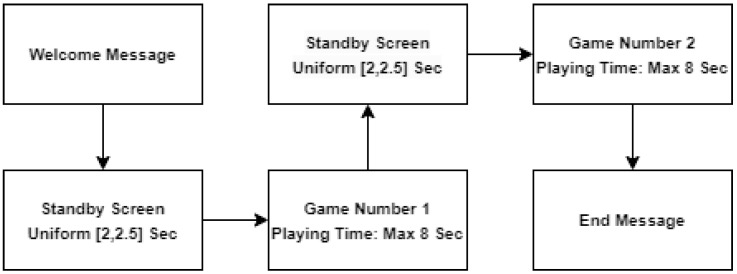
Timeline of experimental stages. The flowchart outlines the sequence of the experimental stages, with slide durations noted for standby and game phases.

Standby slides were displayed for a uniformly random duration between 2 and 2.5 s, and task slides with the word sets were visible for up to 8 s (Figure 2). The participants advanced through the trials at their own pace with a button press, and the order of task trials was randomized across sessions. Their selections were collected in a log file that was continuously updated during the session. The log file also contained temporal information regarding the appearance time of the stimuli, stand-by screen duration, and the time until the participants selected the word. 

Crucially, the participants were informed of a points-based reward system, with 100 points awarded for each word picked in the initial task and for each successful coordination in the subsequent task. However, feedback regarding the success of coordination with their partner was withheld until the end of the experiment, where the rewards were calculated based on random matching with another participant’s choices. This design choice was intended to simulate real-world decision-making scenarios where the immediate outcomes of cooperative efforts are not always evident. In addition, each participant underwent a preliminary training session (that included five coordination problems) to avoid failures resulting from lack of familiarity with the experimental application. The detailed procedural timeline, including the sequence and duration of the different stages, is illustrated in Figure 2, ensuring a clear understanding of the experimental flow and temporal structure.

#### 2.2.3. EEG Recordings and Data Pre-Processing

EEG recording was accomplished with g.USBAMP, an active EEG amplifier with 16 channels manufactured by g.tec, Schiedlberg, Austria. The location of the electrodes was set according to the 10–20 international system, and the sampling frequency was 512 [Hz]. The electrode impedance did not exceed 5 kΩ and was monitored using OpenVibe (Version 3.5.0) [20] recording software.

The EEG data underwent several preprocessing steps, which were successfully implemented in previous studies (e.g., [3,8]). These steps included applying band-pass filtering (BPF) in the range of [1, 32] Hz to capture the delta, theta, alpha, and beta frequency bands. These bands are crucial for the cognitive tasks under investigation and are less susceptible to high-frequency noise [21,22]. After band-pass filtering, artifacts were removed using Independent Component Analysis (ICA), followed by re-referencing the average reference, baseline correction, and downsampling from 512 Hz to 64 Hz. Analysis was conducted on 1 s epoch windows starting from the initiation of each task, ensuring a focus on the most relevant EEG data for the task performance.

#### 2.2.4. Modeling Electrophysiological Spatial Coherence Similarity

Our goal in this section is to describe the analyses stages leading to the construction of our prediction model. The model estimates the similarity between the spatial electrophysiological coherence patterns of two players and predicts, based on the distance between them, whether successful coordination was achieved or not. Using this model, we could examine the effect of the coordination difficulty on the model’s performance. 

**Figure 3:**
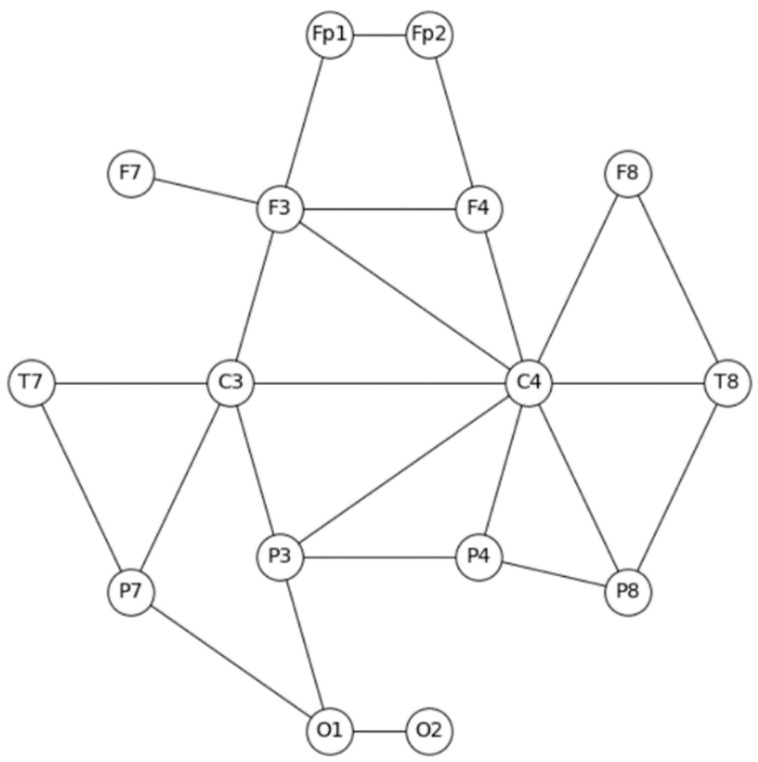
Coherence graph visualization. Graph layout is in accordance with electrode placement on scalp.

The analysis of the raw EEG data comprised three main stages. In the first stage, the EEG epochs underwent preprocessing to improve the signal quality (see Section 2.2.2). In the second stage, we extracted coherence graphs (Figure 3) out of the EEG multichannel record. The EEG coherence measures the level of synchronization between two brain regions of the same person, or alternatively, the compatibility of the brain activity of the same area between two different people [23,24]. Specifically, in the second stage, we computed the coherence (ranging from 0 to 1) for each pair of electrodes present in the electrodes array. The coherence calculation was performed on all the 1 s epochs for each participant. To present the results as a graph, we employed discretization. Specifically, we considered that synchronization existed between electrode pairs if the absolute coherence value was ≥0.5; otherwise, there was no synchronization. 

By setting the threshold at 0.5, we aim to achieve a balance between the sensitivity (the ability to correctly identify true synchronization) and specificity (the ability to correctly identify the lack of synchronization). This balance is essential to ensure that the synchronization we observe is not due to random fluctuations in the EEG signal, but is likely associated with the cognitive phenomena under investigation. In our study, the adoption of a 0.5 threshold for coherence calculations draws upon methodologies used in similar EEG research, notably the approach proposed by Berchicci et al. (2015) [25] and implemented in [26]. Their method involves thresholding the coherence matrix based on its own value distribution, which is typically non-Gaussian in EEG signals. This approach calculates the Median and Median Absolute Deviation (MAD) of the coherence value distribution, retaining only those coherence values that exceed the threshold of Median + 1 MAD as meaningful functional connections.

While our study uses a fixed threshold of 0.5, this choice is conceptually aligned with the approach by Berchicci et al. in prioritizing statistical robustness and significance in coherence analysis. The 0.5 threshold in our study serves a similar purpose: to discern meaningful functional connections in the EEG data by filtering out values that likely represent random fluctuations or noise. A fixed threshold like 0.5 allows straightforward interpretation and comparison with other studies, especially when dealing with complex multi-participant tasks and data that are prone to variability [27]. Furthermore, this threshold is chosen for its practicality and ease of comparability. It provides a clear, replicable criterion for identifying significant functional connections, which is crucial for the robustness and reliability of our findings.

The resulting graph is undirected, which is a type of graph where the edges between the nodes have no specific direction or orientation. This representation is chosen to reflect the symmetric nature of EEG coherence, which indicates the level of synchronization between the electrode pairs without suggesting a directional influence. In our context, the undirected graph effectively captures the bidirectional or symmetrical relationships in the brain activity, which are essential for understanding the neural dynamics of coordination tasks. The adjacency matrix, which is symmetric and has dimensions of 16 × 16, represents the connections between the electrodes. Each graph (i.e., each epoch) can have a maximum of 120 edges, which is calculated as ($\frac{n*(n-1)}{2})$, where n is the number of nodes. Each node in the graph, such as an electrode, can have a maximum of 15 edges, excluding the self-loops. The self-loops were excluded because the coherence value between a signal and itself is always 1. As an example, let us consider the Fp1 electrode in a single epoch of coordination. Fp1 exhibited an absolute coherence value ≥ 0.5 with Fp2 and F3. Therefore, the nodes representing these electrodes are connected to the Fp1 node.

The resulting spatial coherence graph enables the comparison between the responses of the two players, while attempting to coordinate.

In the third stage, we converted each coherence graph into a feature vector that preserved the spatial structure using an anonymous random walks embedding scheme. Anonymous random walks graph embedding is a method that transforms graph information into numbers. It achieves this by simulating random walks on the graph, where we start at a node and randomly move to its neighboring nodes. These random walks help us understand the graph’s structure and connections. After repeating this process several times, we create sequences of visited nodes during the walks. These sequences are then converted into numerical vectors based on the probability density functions of the walk’s paths. These vectors represent the nodes in a manner that maintains their relationships and can be utilized for different machine learning tasks, like classifying nodes or predicting links [8,13]. To implement the optimal embeddings, we performed a grid search on several random walk lengths, which were estimated according to the classification results that will be presented later. A random walk with a walk length of five produced the optimal results.

**Figure 4:**
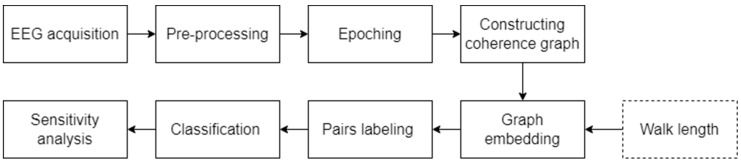
Experimental scheme.

After a feature vector represented each graph, in the fourth stage, we labeled each possible pair of players. Since there were 10 players, the number of different pairs that can be produced is 45. Since each pair played 12 coordination games, there were 540 observations in total. The labelling process of pairs of players was conducted as follows. We calculated the absolute difference between the vectors for each pair of players for the same game board. If the two players managed to coordinate on a specific board, we labeled the vector as “1”; if they failed to coordinate, we labeled it as “0”. Out of the 540 total observations, 153 were positive instances of successful coordination, in which players managed to converge on the same answer, and 387 were negative instances where players failed to coordinate. Subsequently, all the samples were entered into the XGBoost model (e.g., [28,29]). To avoid over-fitting, we worked with a three-fold cross-validation method, and each fold was equally distributed between the classes. For each fold training session, we used the same XGBoost hyperparameters (i.e., the number of estimators and maximal depth). The entire experimental process, from the acquisition of the EEG signals to graphic representation and up to the stages of labeling and model training, is described in Figure 4.

## 3. Results and Discussion

The performance of the classification model was gauged using three key metrics: precision, recall, and the F1 score. Precision quantifies the model’s accuracy in identifying the true positives for coordination, while recall measures the model’s ability to capture all the relevant instances of coordination. The F1 score, a composite metric, conveys the balance between precision and recall, providing an overall performance indicator. These metrics are pivotal for understanding the model’s effectiveness in discerning the EEG patterns associated with coordination.

### 3.1. Model Performance Metrics

The model’s performance, articulated through sensitivity variables, is a testament to its capability in discerning true coordination events within the EEG data. With an accuracy of 85.19% (460/540), the model demonstrates high precision, especially in identifying a lack of coordination, which is 91.15% for label “0”. For predicting coordination (label “1”), the model maintains a precision of 71.86%. The recall values are similarly encouraging, with 87.86% for label “0” and 78.43% for label “1”.

### 3.2. Classification Performance as a Function of Coordination Difficulty

As we probe further into the impact of the coordination game’s difficulty level, represented by the CI values, we see a nuanced effect on the classification performance. The CI, quantifying the frequency of successful coordination at the group level, provides a measure of the complexity inherent in each game. It thus serves as a critical variable in our analysis, representing how the model performs across different levels of task difficulty. The previous studies have shown that the level of difficulty of the coordination game, which is derived from the saliency of the focal point, affects the players’ behavioral responses [1] and the accompanying electrophysiological patterns (e.g., [30]). The saliency of the focal point of our study refers to how prominently a particular choice or option in the tacit coordination game captures the players’ attention, guiding their decision making towards synchronized selections without direct communication.

The variability in the CI across different games reveals the range of difficulties the participants encountered. Our analysis assesses how this variability relates to the model’s precision and recall, thus informing the robustness of our classifier at varying levels of coordination complexity. The purpose of this section is to examine the effect of the difficulty level of the coordination game on the performance of the electrophysiological classifier described in Section 3.1. That is, we would like to figure out whether the difficulty level of coordination affects the spatial coherence patterns that constitute the input of the model.

Since, in this experiment, all the participants played the same set of coordination games, we wanted to quantify the difficulty level of each of the different games. To do so, we used the CI [15,16]. The CI estimates the actual frequency of successful coordination at the group level, and therefore, reflects the degree of complexity required to achieve successful coordination in each game. Based on the answers of all ten players, we calculated the CI value for each of the 12 coordination games used in the study (see Table 1).

The distribution of the CI values indicates that there is a significant variation in the difficulty levels between the different games. The easiest game to coordinate was game #7 with a CI of 0.444, while the two most difficult games to coordinate were games #4 and #9, each with a CI of 0.178. This means there is a gap of about 250% between the hardest and the easiest games. In other words, the probability of successful coordination in the easiest game is 2.5 times greater than that in the hardest game. To assess the relationship between the performance of the classifier and game difficulty, we calculated the values of precision, recall, and F1 score for each of the twelve games (Table 2).

**Figure 5:**
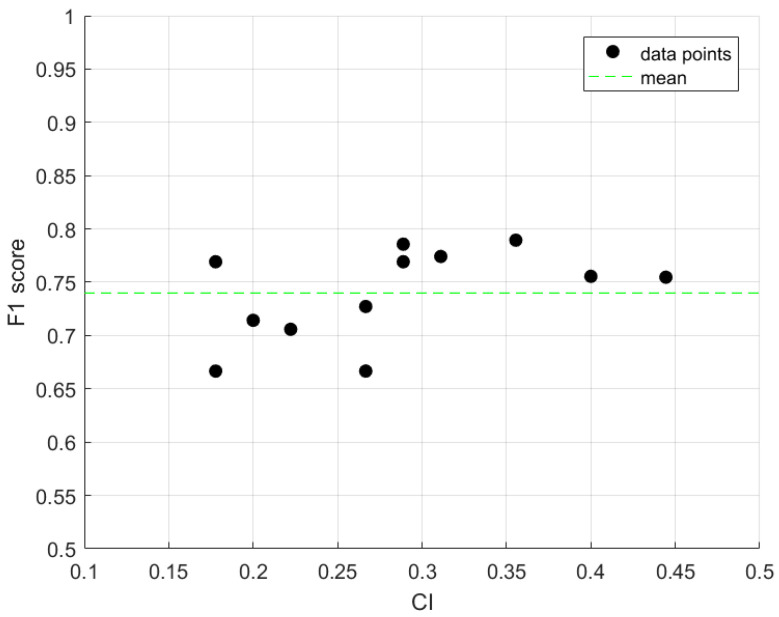
F1 score vs. coordination index (CI).

Subsequently, we plotted the F1 score as a function of the game difficulty, as reflected by the CI (Figure 5). The dispersion of the values is relatively narrow, as the difference between the minimum and maximum values is only 15.46%. Accordingly, there is no substantial linear relationship (i.e., correlation) between the CI and the F1 score.

**Figure 6:**
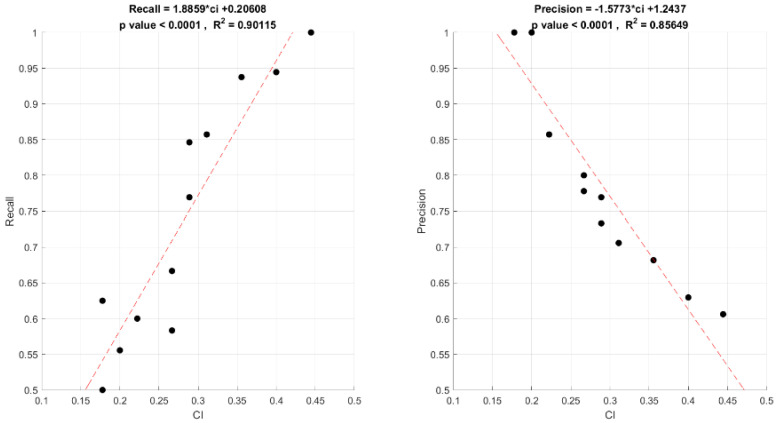
Precision and recall as a function of the coordination index (CI).

Thus, apparently the CI does not affect the classifier’s performance. However, we further examined the relationship between the CI and the two model evaluation metrics, precision and recall, respectively (Figure 6). A statistically significant linear relationship was found between the CI and both the precision and recall (*p* < 0.0001). That is, the CI value significantly affects the model’s performance, which is based on the players’ spatial coherence patterns. 

Noteworthily, the relationships of the CI with recall (Figure 6, left panel) and precision (Figure 6, right panel) present opposite trends. As the CI value increases, i.e., as the game is easier to coordinate, the recall value increases. That is, the probability of identifying a pair of EEG epochs, reflecting successful coordination from the pool of all the relevant cases, increases with the CI value. In the case of precision, the trend is exactly the opposite. As the CI value increases, the precision value decreases. That is, the easier the game is to coordinate, the more false positives there are (predicting successful coordination, while, in fact, it was unsuccessful).

Our sensitivity analysis, as reflected in Figure 5 and Figure 6, sheds light on the distinct behaviors of the model’s performance metrics in relation to the difficulty of the coordination tasks. Figure 5 indicates that the F1 score, which combines the precision and recall, does not show a strong correlation with the coordination index (CI), signifying that the model’s accuracy is consistent across tasks of varying complexity. Figure 6 provides a more detailed view, showing divergent trends for precision and recall with increasing CI values. Specifically, as the CI rises, suggesting simpler tasks, the model’s recall improves, pointing to a higher likelihood of identifying true coordination. In contrast, the precision decreases as the CI increases, suggesting a rise in false positive rates. This sensitivity analysis highlights the importance of considering both precision and recall, which together offer a comprehensive view of the model’s performance and its ability to identify true coordination events against the backdrop of the tasks’ cognitive demands.

## 4. Conclusions and Future Work

Our study’s goal was to develop a machine learning model capable of predicting coordination through EEG spatial features. We found that the model’s sensitivity to detecting coordination is not solely dependent on the CI. The complexity of the coordination task, as quantified with the CI, does indeed impact the model’s precision and recall in a meaningful way. As the tasks become less complex, the likelihood of successful coordination detection increases; yet, this also leads to a higher rate of false positives. This interesting inverse relationship between precision and recall relative to the task difficulty highlights the interplay between the classifier’s performance and the cognitive demands of the tasks. Our analysis, which revealed a nuanced relationship between the model’s precision and recall in relation to the CI, underscores the complexity inherent in predicting coordination through the EEG spatial features. This leads us to revisit and emphasize the core objective of our research.

The primary aim of this study was to construct a machine learning model capable of discerning the similarities between spatial EEG features during coordination processes. This goal was pursued with a keen focus on how the model responds to varying levels of task difficulty, as quantified with the CI. Here, we provide a detailed analysis, demonstrating the model’s sensitivity, which was assessed through three conventional measures: precision, recall, and the F1 score. These metrics, which were evaluated in the context of the coordination task’s difficulty, offer a comprehensive view of the model’s ability to predict whether two players are likely to converge on the same solution based on the similarity in their EEG spatial coherence patterns.

The coherence patterns were represented by an undirected graph with 16 nodes, which was embedded using an anonymous random walk with five steps before being predicted with the model. We analyzed the model’s performance as a function of task difficulty, which was measured with the CI (i.e., the coordination index). Specifically, we calculated the regression between the CI and the corresponding precision and recall values. A statistically significant relationship was found between the CI value and both the precision and recall. However, whereas recall was positively correlated with the CI, an inverse correlation was found between precision and the CI. Thus, as the game becomes easier to coordinate, the precision decreases (the number of false alarms increases), but, in contrast, the recall increases (the probability of detecting a true coordination event increases).

We then analyzed the findings considering the game theory. When the game is easier to coordinate (i.e., a higher CI), the focal point solution [2] stands out more clearly, and this saliency of the focal point elicits a similarity between the two compared EEG patterns. Hence, the focal point increases the chances of discovering successful coordination using the predictive model, and the recall value increases. At the same time, the players who did not end up choosing this focal point solution for various (e.g., strategic) reasons, were, nevertheless, exposed to its saliency and were, therefore, affected by its prominence; although, they eventually chose different solutions. This evoked similar EEG patterns in both players, and consequently, more false positive errors, which caused a decrease in the precision value, as presented in Figure 6.

Recent studies (e.g., [31,32]) have explored innovative approaches in EEG analysis and machine learning, such as Convolutional Neural Network–Long Short-Term Memory (CNN-LSTM) and the Spiking Neural Network (SNN). While these methods offer valuable insights, they are particularly tailored to addressing specific methodological challenges, such as time series analysis and noise robustness.

In contrast, our study was tailored to addressing the unique challenges of coordination processes, focusing on unravelling the nuances within EEG data, specifically within the context of spatial coherence. EEG data often exhibit intricate relationships among the electrodes, and the random walk method we employed excels in capturing these intricate spatial coherence patterns. Its strength lies in identifying subtle connections and dependencies among the EEG nodes, which is a fundamental aspect for comprehending coordination dynamics.

In conclusion, while we acknowledge the value of the alternative methods, the random walk algorithm was intentionally chosen for its unique capability to capture the intricate spatial coherence patterns within the EEG data, aligning with our study’s core focus on coordination processes.

This study has several limitations that should be considered. First, the group of participants in the experiment is homogeneous. The participants have the same cultural background, education, and other demographic data, which may affect both the strategic behavior and the perception of the game’s difficulty level [33]. Second, the EEG acquisition system included 16 electrodes. A higher number of electrodes, for example, 32 or 64, could produce spatial coherence graphs with a higher resolution. A graph with a higher resolution may have allowed the modeling of more complex maps, which may be required in different scenarios such as complex games. Finally, all the coordination games in this study are based on semantic decision making. Therefore, our results should be validated in cases where the decision making is based on different features, such as size, shape, color, etc.

Our results introduce several possibilities for future studies. First, it would be interesting to examine whether the level of task difficulty also affects the performance of the classifier, which is based on spectral features [3]. Second, it will be interesting to examine whether it is possible to use the effect of the CI on classifier performance, which is based on the EEG signals, to design an agent that optimizes the coordination results (e.g., [34,35,36,37]). Third, the future research could benefit from a comparative analysis involving both connectivity and spectral measures to enrich the understanding of the EEG patterns in coordination tasks. Furthermore, it would be valuable to explore the connection between the CI and spatial coherence in different contexts, such as employing wider CI ranges and alternative coordination mechanisms. Finally, the previous studies have shown that several additional parameters, such as social value orientation [38], loss aversion [39], and culture [33], influence a player’s behavior in coordination games. Thus, it will be worthwhile examining their interaction with the CI and their effect on the classifier performance in subsequent efforts.

## Figures and Tables

**Table 1:** CI distribution over experiment games.

**Game**	1	2	3	4	5	6	7	8	9	10	11	12
**CI**	0.311	0.266	0.266	0.178	0.356	0.222	0.444	0.289	0.178	0.289	0.200	0.400

**Table 2:** The performance of the classification model in each game.

Game	1	2	3	4	5	6
CI	0.311	0.266	0.266	0.178	0.356	0.222
Recall	0.857 (12/14)	0.583 (7/12)	0.667 (8/12)	0.500 (4/8)	0.937 (15/16)	0.600 (6/10)
Precision	0.706 (12/17)	0.778 (7/9)	0.800 (8/10)	1.000 (4/4)	0.682 (15/22)	0.857 (6/7)
F1 score	0.774	0.774	0.727	0.667	0.789	0.706
**Game**	**7**	**8**	**9**	**10**	**11**	**12**
CI	0.444	0.289	0.178	0.289	0.200	0.400
Recall	1.000 (20/20)	0.846 (11/13)	0.625 (5/8)	0.769 (10/13)	0.556 (5/9)	0.944 (17/18)
Precision	0.606 (20/33)	0.733 (11/15)	1.000 (5/5)	0.769 (10/13)	1.000 (5/5)	0.623 (17/27)
F1 score	0.755	0.786	0.769	0.769	0.714	0.756
**Full model** Recall: 0.784 (120/153); Precision: 0.718 (120/167); F1 score: 0.750						

## Data Availability

All the experimental data, which include the players’ electrophysiological recordings and the corresponding coordination logs, are stored on the servers of Ariel University. The data can be obtained by request from the IRB member, Chen Hajaj (chenha@ariel.ac.il), or from one of the authors (Dor Mizrahi—dor.mizrahi1@msmail.ariel.ac.il; Ilan Laufer—ilanl@ariel.ac.il; Inon Zuckerman—inonzu@ariel.ac.il).
